# A New Way to Treat Central Nervous System Dysfunction Caused by Musculoskeletal Injuries Using Transcranial Direct Current Stimulation: A Narrative Review

**DOI:** 10.3390/brainsci15020101

**Published:** 2025-01-21

**Authors:** Stéphane Perrey

**Affiliations:** EuroMov Digital Health in Motion, University of Montpellier, IMT Mines Ales, 34090 Montpellier, France; stephane.perrey@umontpellier.fr

**Keywords:** sports, pain sensitivity, motor performance, ankle instability, knee injuries, neural drive, excitability, recovery, central nervous system

## Abstract

Background: Musculoskeletal injuries can have far-reaching consequences on brain function, leading to reduced motor control, altered movement patterns, increased inhibition of the injured muscle and joint, and changes in neuroplasticity. These deficits, controlled in part by the central nervous system (CNS), might be alleviated with an appropriate adjuvant treatment. One possibly suited treatment at the CNS level is transcranial direct current stimulation (tDCS), which modulates cortical excitability and further neuroplasticity. Objectives: The present review outlines the multifaceted repercussions of common musculo-skeletal injuries on CNS functions and presents original studies that mostly report beneficial effects regarding the use of the tDCS intervention in people who had experienced musculoskeletal injury rehabilitation. Results: The first evidence suggests that tDCS, targeting brain areas responsible for motor control or on sensory and pain-related brain regions, may offer significant benefits in the recovery of brain function and motor performance following musculoskeletal injuries. Key findings include enhanced motor function, altered CNS excitability and inhibition, and reduced pain perception, all contributing to improved rehabilitation outcomes. However, the paucity of studies and the heterogeneity of injuries render it challenging to ascertain the optimal treatment parameters. Furthermore, the variability regarding stimulation parameters is a crucial aspect that remains to be addressed and limits the possibility of generalizing these first findings. Conclusions: It is concluded that well-powered trials with standardized protocols should be conducted to confirm these effects and establish clear clinical guidelines for the use of tDCS in sports injury rehabilitation.

## 1. Introduction

Muscle injuries, whether acute (e.g., strains, sprains) or chronic (e.g., tendinopathies), are common among both athletes and the general population. Anterior cruciate ligament (ACL) injuries are among the most common sports-related injuries [1]. The general population and athletes may also experience ankle sprains, which lead to reinjury and the formation of chronic ankle instability (CAI) [2]. Patellofemoral pain (PFP) appears prevalent throughout the lifespan, affecting not only the general population, but also highly active individuals [3]. Finally, sports that involve jumping, twisting, collisions with other players, and quick direction changes are the most likely to cause injuries that lead to knee osteoarthritis (KO, a painful chronic joint disease leading impairment of physical function) [4]. These common musculoskeletal injuries can lead to substantial functional impairments including muscle weakness, limited range of motion, and prolonged recovery times [5]. They are also known to be associated with a long-lasting inability to fully activate the muscle, a process known as arthrogenic muscle inhibition (AMI) that significantly dampens recovery [6]. In addition, these injuries can have significant effects on brain function, especially in areas responsible for motor control, movement planning, and motor inhibition [7]. The motor cortex and other brain regions involved in voluntary movement can be directly influenced by musculoskeletal injuries, leading to changes in how the brain processes motor information, coordinates muscle actions, and regulates movement [8]. These consequences can affect both motor performance and neuroplasticity (i.e., the brain’s ability to reorganize itself after injury) [8,9]. These functional deficits, controlled by the central nervous system (CNS), may contribute to poor long-term outcomes in people who have sustained musculoskeletal injuries to the lower limbs [7]. In the realm of sports injury rehabilitation, addressing CNS dysfunction is crucial for optimal recovery.

Traditionally, rehabilitation strategies for musculoskeletal injuries have focused on physical therapy, rest, pharmacological treatments, and increasingly, advanced neuroplasticity-based interventions [10]. Among the latter, emerging research suggests that non-invasive brain stimulation techniques, particularly transcranial electrical stimulation (tES), could play an important role in enhancing recovery during musculoskeletal injury rehabilitation by modulating the primary motor cortex (M1) function [11]. tES affects the brain states through different current waveforms (direct, alternating, random noise stimulation) applied transcranially. This form of neuromodulation uses low electrical current to modulate cortical excitability and neuronal activity, and has shown promise in improving motor function, pain management, and neuroplasticity [12], which could be particularly useful in the context of musculoskeletal injury rehabilitation [13]. The advantages of tES including its cost-effectiveness, safety, portability and relative ease of use have resulted in a significant increase in its utilization in research and clinical settings [14].

Among the tES techniques, transcranial direct current stimulation (tDCS) has given rise to a heightened level of interest in non-invasive brain stimulation, primarily due to its ease of use and its capacity to produce significant effects on human neural plasticity [11,15]. This most widely used form of tES involves applying a low direct amplitude current (typically 1–2 mA) through two or more electrodes placed on the scalp to create a weak electric field (<1 volt per meter) in the brain, with minimal side effects (e.g., itching, burning sensation, or headache) [12,15]. The anodal electrode is typically placed over M1, which is responsible for motor control, while the cathodal electrode may be placed over the contralateral cortex or a different region, depending on the desired outcome [14,15]. In brief, the stimulation modulates neuronal activity by either increasing (anodal) or decreasing (cathodal) the excitability of the targeted brain regions [12]. The physiological mechanisms of tDCS to enhance motor performance were addressed in one recent review [16]. tDCS works by changing the resting membrane potential of cortical neurons, and can modulate the cortical excitability to find out the neuro-physiological functions in some brain regions such as M1 [17] or the prefrontal cortex (PFC) [18], being used in many different domains for the recovery of functional performance during musculoskeletal injury rehabilitation.

Despite the extensive range of applications of tDCS in the fields of exercise, rehabilitation medicine, and sport science, there is a paucity of data regarding the CNS responses to musculoskeletal injuries driven by tDCS intervention. The findings of two reviews and meta-analyses [19,20], encompassing up to 14 studies involving 740 patients with KO, indicated that tDCS may serve as a viable pain management approach for individuals with KO. A recent systematic review with a meta-analysis suggested that several weeks of tDCS may also be effective to counteract AMI [21]. As such, the present narrative review aims to explore the current evidence on the potential effects of tDCS on the CNS function after common musculoskeletal injuries, specifically its influence on motor performance recovery, neuro-plasticity, and pain management. The initial section delineates the repercussions of musculoskeletal injuries on the CNS functions, while the subsequent section concentrates on the controlled studies employing tDCS in musculoskeletal injury rehabilitation to augment both motor and CNS functions, along with pain sensitivity.

## 2. The Multifaceted Repercussions of Musculoskeletal Injuries on the Central Nervous System

**Figure 1:**
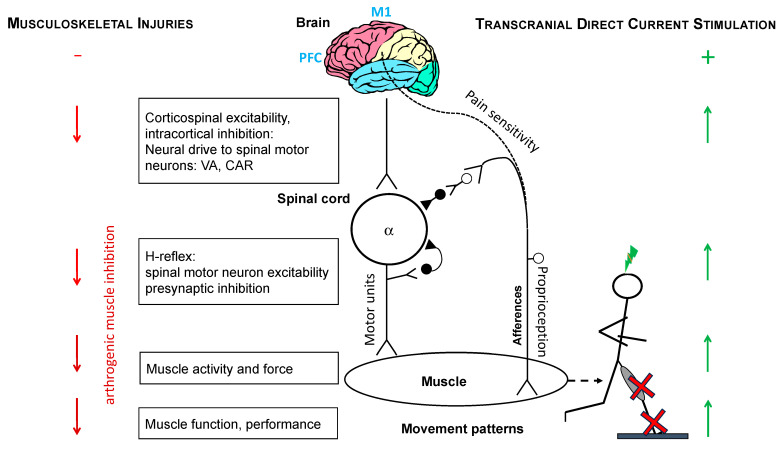
Potential sites (brain, spinal cord, muscle, and movement patterns) that can be altered by the occurrence of musculoskeletal injuries (**left side**) or following the intervention of transcranial direct current stimulation (**right side**). Their opposite effects are addressed in Section 2.1, Section 2.2, Section 3.1 and Section 3.2. The vertical arrows denote the direction of changes (increase, decrease) for each site. The cross (red) shows musculoskeletal injuries to the lower limbs, and the lightning bolt (green) represents the transcranial electrical stimulation add-on when moving. Dashed line symbolizes neural pathways between proprioceptive feedback from the muscle and surrounding and the brain (including the somatosensory cortex). The lower motor neuron (alpha α) innervates the effector muscle(s). A reflex arc (monosynaptic or polysynaptic) allows for interpretation and response to musculoskeletal damage directly through the spinal cord, bypassing the brain. VA, voluntary activation; CAR, central activation ratio; H-reflex, Hoffman-reflex; M1, primary motor cortex; PFC, prefrontal cortex.

The influence of typical sports injuries (e.g., strains, sprains, swelling, tear, tendinopathies) on muscle function can be understood through several key mechanisms. Following an injury, muscles can undergo atrophy due to disuse, resulting in weakness and a reduction in muscle size [22]. This phenomenon is particularly evident in muscles that are immobilized due to fractures, sprains, or tendon/ligament injuries. Even after an injury has healed, muscles may not fully regain their pre-injury force production levels [23]. This is due to the fact that muscle fibers and motor units (groups of muscle fibers controlled by a single motor neuron) may require time to recover or may remain impaired. In addition, many sports injuries, such as ligament sprains or fractures, can result in joint stiffness and a limited range of motion. This can affect the way muscles contract and lengthen, impacting overall muscle function. Following injury, the formation of scar tissue around the affected area can reduce muscle elasticity and flexibility, thereby limiting full muscle extension and contraction [24]. Musculoskeletal injuries can have significant consequences not just on the muscle tissue itself and surrounding tissues, but also on the CNS [7,8,9,25], which controls and coordinates muscle function. The CNS including the brain and spinal cord plays a critical role in regulating muscle movements and responding to musculoskeletal injury (see Figure 1). Several key consequences of common musculoskeletal injuries (ACL, CAI, KO, PFP) from the literature on the CNS and compensatory adaptations are presented below.

### 2.1. Altered Neural Control and (Mal)Adaptative Changes

Following a musculoskeletal injury, especially one that involves pain or prolonged disuse, the CNS may reduce its neural drive (the communication from the brain to the muscles), resulting in decreased muscle activation and compromised movement control [26,27,28]. Methodological assessments of neural activity in the injured limb include the central activation ratio and electromyographic responses. Muscle activation failure is often seen with injury-related muscle weakness, as the nervous system might not fully activate the injured muscle due to pain or swelling [7]. In this case, the number of motor neurons available for recruitment is often reduced (i.e., a smaller motor neuron pool excitability), and the voluntary recruitment of motor neurons to a normal extent is diminished (i.e., central activation failure) [29]. Then, the motor cortex has difficulty issuing coordinated, fluid motor commands to the injured muscle or compensatory muscles, which can result in poor muscle function or motor performance, with the muscle being less efficient in generating force [30].

However, the CNS has a huge adaptive ability known as neuroplasticity, which allows the brain to reorganize its neural circuits in response to injury [25,31]. Indeed, it can adapt to a musculoskeletal injury by developing new movement patterns to compensate for the loss of function in the injured muscle/joint/tendon or ligament. For instance, individuals may utilize the contralateral limb or other muscles in an inefficient manner, which the brain subsequently attempts to coordinate. While this compensatory movement can help maintain some level of activity, it can lead to abnormal motor patterns, placing stress on other muscles and joints and increasing the risk of further injury. It is important to note that, over time, this can become ingrained as a compensatory motor pattern. This can affect, in turn, posture and coordination, leading to imbalances or overuse injuries in other parts of the body. In this case, this reorganization results in maladaptive changes [7], leading to inefficient movement patterns or diminished motor control. In addition to the recruitment of muscles not typically involved in movement, which is a compensatory mechanism for an injured muscle, muscle injuries have been shown to impede the CNS’s capacity to restore full coordination and motor control [32,33]. This effect can be particularly pronounced in activities that require fine-motor skills or rapid, complex movements such as running, jumping, and fine-motor tasks. This is because the CNS may need to retrain itself to compensate for the injury and restore the neural pathways responsible for smooth and efficient movement.

### 2.2. Altered Cortical Inhibition, Spinal Reflexes, and Reduced Proprioception

Injury-induced inhibition can lower the excitability of M1, leading to reduced neural drive and less efficient muscle contraction [28,34]. As a result, the brain may struggle to produce the same level of force or endurance in the injured muscle, even after physical recovery. After a muscle injury, the motor cortex tends to exhibit increased inhibition toward the injured muscle to prevent further damage. This protective mechanism, known as inhibitory control and clinically termed AMI (Figure 1), reduces the activation of the injured muscle to avoid strain or reinjury [6]. However, excessive inhibition can persist even after the tissue has healed, resulting in profound muscle weakness [35] or postural control deficits [36]. Chronic pain or disuse of an injured limb may lead to an imbalance in cortical inhibition [37], where the brain fails to sufficiently activate the injured muscle or other muscles in the same motor group. This can prevent a smooth and coordinated recovery process, causing long-term deficits in strength and movement control [38].

The muscle inhibition responsible for the aforementioned observations is reflexive and mostly happens in the spinal cord (lesser spinal-reflexive excitability utilizing the Hoffmann reflex) [36]. The injury to muscles and their associated nerves may impair reflex actions that the CNS normally regulates [34,36]. For example, if an injury causes significant pain or swelling, the reflexive response to protect the injured muscle may become exaggerated or delayed. In turn, this can alter muscle function and result in an ineffective defense mechanism (e.g., the withdrawal reflex or postural adjustments). The injury may lead to abnormal responses from the spinal reflex pathways, disrupting normal motor function [37]. The extant literature appears to demonstrate a certain degree of consistency with regard to the early changes in the spinal reflex excitability of corresponding muscles following the initial joint injuries. These changes are likely to be due to any combination of tissue damage, joint laxity, effusion, pain, or inflammation [35,39].

Impairment of physical function due to musculoskeletal injuries at the lower limbs creates joint instability at both the ankle and knee, increasing the joint loading that contributes to balance deficits [36], a degradation of movement, and possible reinjury [7]. Finally, when a muscle is injured, proprioceptive feedback from mechanoreceptors and/or diminished postural reflex responses and surrounding tissues may be impaired, leading to poor coordination and reduced control over movements [40,41]. Proprioception refers to the ability to sense the position of the body parts and how they move in space. This is especially important in activities that require fine-motor skills or balance, as the CNS may struggle to accurately sense muscle tension or joint position. Injuries can also affect kinesthetic awareness, which is the sense of how your body is moving in real-time. Musculoskeletal injuries may disrupt the body’s ability to coordinate movements smoothly, resulting in difficulty in performing complex motor tasks or movements that require fine control [42].

### 2.3. Increased Pain Sensitivity (Central Sensitization)

Musculoskeletal injuries, especially severe (or chronic) ones, can lead to changes in the way the CNS processes pain [43]. This phenomenon is known as central sensitization, defined as an amplification of neural signaling within the CNS that elicits pain hypersensitivity [44]. This can lead to an exaggerated pain response, even from minor stimuli or normal movement, causing a cycle of ongoing pain and reduced muscle function. In this case, the motor cortex may become more sensitive to input from pain receptors in the injured muscle, which can impair motor control and lead to dysfunction in motor function. A condition resulting from central sensitization, hyperalgesia refers to an increased sensitivity to pain. The CNS becomes more responsive to pain signals, resulting in heightened pain perception and discomfort, even after the injury has started to heal. In other words, the pain processing centers in the brain (including the somatosensory cortex) may become hyperactive, reducing the brain’s ability to focus on motor control, leading to changes in muscle activation patterns. As a result, individuals may experience involuntary muscle guarding or increased tension in the injured muscle(s), further impeding normal movement.

The stress and pain of a musculoskeletal injury can also affect cognitive function, particularly attention and focus. This can make it more difficult for the brain to properly coordinate muscle movements or maintain consistent training efforts during recovery. Injuries to muscles/joints, particularly when pain or prolonged recovery is involved, can place an additional cognitive load on the brain. The brain has to process not only the physical rehabilitation and movement control, but also the psychological effects of the injury, such as anxiety, frustration, and fear of re-injury, which can lead to avoidance behaviors or an overly cautious approach to rehabilitation. This can further affect motor control by diverting attention away from movement execution. Persistent pain resulting from musculoskeletal injury can lead to long-term changes in motor function and abnormal patterns of muscle activation, and can have long-term effects on brain regions involved in both pain perception and motor control. Brain areas like the PFC, which is responsible for decision-making and goal setting, may become hyperactive or underactive due to chronic pain [45], leading to reduced cognitive flexibility and impaired motor function.

## 3. Effectiveness of Transcranial Direct Current Stimulation in Musculoskeletal Injuries Rehabilitation

A literature search was performed in the PubMed database and the search engine Google Scholar using the keywords “transcranial direct current stimulation” OR “tDCS” AND “muscle” OR “musculoskeletal” AND “injury”. Only original studies published in the English language were reviewed and included in the present review. Additional articles were identified via recursive reference searching and previous knowledge. For the purposes of this review, intervention studies (controlled trials) were included if they investigated the use of tDCS to treat muscle or musculoskeletal injuries in humans. The reference lists of the included studies were also scanned to generate a broader scope of the search. Research articles published on the application of tDCS on spinal and brain disorders were excluded. The review of the extant literature revealed a paucity of studies in the area of tDCS and musculoskeletal injuries, with the majority focusing on athletes or healthy individuals following common sports injuries such as tendon injuries, joint sprains (CAI, KO, PFP), and ligament damage (ACL). The sample sizes of these studies ranged from 10 to 80 participants (see Table 1). The studies were principally conducted in sports medicine clinics, rehabilitation centers, and academic research settings. The majority of studies applied anodal transcranial direct current stimulation (tDCS) to the motor cortex (M1), while others targeted sensory or pain-processing areas of the brain (PFC).

### 3.1. The Promises/Benefits of Transcranial Direct Current Stimulation

The effectiveness of tDCS in enhancing motor function during recovery largely depends on the stimulation parameters (e.g., intensity, duration, frequency, timing, and location of stimulation), which are carefully tailored to optimize the desired effects. Research has shown that these parameters can be adjusted to promote neuroplasticity and long-lasting changes in brain networks, potentially outlasting the stimulation period [56,57].

To maximize the benefits of tDCS for motor function recovery, several strategies can be implemented to identify and refine the appropriate stimulation parameters. Here are some key considerations for optimizing the tDCS-induced effects. Stimulation over M1 can enhance motor control in the affected limb. Neuroimaging methods like electroencephalography [58], functional magnetic resonance imaging [59], functional near-infrared spectroscopy [60], and navigation systems can guide optimal electrode placement by identifying specific brain regions that are most relevant to the injured limb or movement dysfunction. In addition to M1, targeting brain areas involved in motor planning, such as the premotor cortex, parietal cortex, and basal ganglia, could enhance recovery. For example, tDCS over M1, combined with premotor cortex stimulation, may enhance motor learning and coordination [61]. The tDCS protocols varied across studies, with most using anodal stimulation (which is thought to enhance cortical excitability) over M1 [12,16]. The stimulation intensity ranged from 1 to 2 mA, with session durations typically lasting 20 min (Table 1). However, individual variability must be considered, and the response to stimulation can depend on factors such as age, baseline motor function, injury type, and pre-existing cortical excitability. Treatment duration ranged from a single session to multiple sessions over a period of weeks, depending on the study design (Table 1). To induce long-lasting changes in motor function, multiple sessions of anodal tDCS over several days (3–5) may be needed [62,63]. This repeated stimulation approach can enhance neuroplasticity and increase the likelihood of inducing long-term effects [63]. Some studies (see Table 1) applied tDCS during rehabilitation exercises (e.g., physical therapy, strength training), while others administered tDCS independently prior to rehabilitation exercises. The effects of tDCS on motor function recovery can be optimized when combined with motor tasks [64]. For example, applying tDCS before or during motor training sessions can prime the motor cortex to be more receptive to rehabilitation efforts, facilitating faster learning and motor function recovery in patients [65]. Note that a protocol combining tDCS with sensory or motor stimulation can enhance neuroplasticity. This method, known as paired associative stimulation, uses brief electrical pulses to stimulate peripheral nerves (e.g., over the injured muscle) while simultaneously applying tDCS to the motor cortex, thereby increasing synaptic plasticity in the motor cortex [66].

### 3.2. Motor Recovery After Musculoskeletal Injuries and tDCS-Induced Neuroplasticity

One of the key domains where tDCS shows promise is in the motor recovery of injured limbs. Musculoskeletal injuries result in motor dysfunction such as weakness, altered movement patterns, and delayed or reduced activation of the injured muscle (see Section 2.1, Figure 1). It is evident that the motor cortex of the brain plays a pivotal role in regulating muscle function. In light of this, a limited number of studies have investigated the impact of transcranial direct current stimulation (tDCS) on the recovery of motor function following musculoskeletal injuries. Anodal tDCS applied over M1 has been shown to improve cortical inhibition [11] and excitability [46] during rehabilitation following knee (ACL) and ankle (CAI) injuries, which can facilitate central motor control and improve muscle strength and motor function. It is thought that maladaptive neuroplasticity is the cause of altered movement patterns. Correction of neural excitability through tDCS should, in turn, improve functional performance outcome (Figure 1). To date, however, there has been a paucity of research in this field, with only a small number of investigations having assessed the effects of tDCS intervention on the functional performance status of people with common musculoskeletal injuries (Table 1). The combination of tDCS with conventional rehabilitation techniques such as physical therapy or strength/resistance training has demonstrated enhanced rehabilitation outcomes in people with CAI, KO, and ACL reconstruction.

CAI is characterized by recurrent ankle sprains with persistent symptoms including pain, recurrent episodes, swelling, limited motion, and weakness [67]. Functional performance and cortical excitability were shown to be improved following 4-weeks of eccentric ankle exercise in combination with anodal tDCS over M1 in individuals with CAI [46]. Based on movement patterns during a drop landing test, Huang et al. [47] showed that tDCS with Bosu ball training had better effects than Bosu ball training in reducing the injury potential during drop landing in people with CAI. Positive effects on proprioception and dynamic balance were reported after an intervention comprising short-foot exercise combined with high definition tDCS [48]. Among the interventions that could treat arthrogenic muscle inhibition in patients with CAI, the systematic review with meta-analysis conducted by Kim et al. [21] suggests that several weeks of tDCS may be effective to counteract arthrogenic muscle inhibition.

The majority of ACL injuries have a non-contact mechanism. The common mechanism of injury is produced with a non-contact valgus force combined with an internal rotation of the tibia. As previously stated, ACL patients demonstrate deficiencies in excitability of the motor cortex. Consequently, ACLR patients may experience benefits from augmented corticospinal activation during exercise via tDCS, in addition to potential mitigations in pain, thus enabling enhanced levels of peripheral muscle activation. The scarce studies found thus far have mitigated these findings. Using a randomized cross-over design, Rush et al. [49] observed that there were no differences in quadriceps muscle activity or any self-reported pain outcomes when comparing single active tDCS and sham treatments. Recently, 6 weeks of anodal tDCS following ACL was shown to reduce quadriceps intracortical inhibition and facilitation but without significant changes in quadriceps voluntary strength [11]. The effects of anodal tDCS M1 concurrent with physiotherapy training (10 sessions) significantly improved the postural control and muscular performance in athletes with an ACL injury until one month after [45]. This effective lasting effect was likely induced by a multi-session protocol and concurrent intervention. In addition, the functional test used in [50] focused on postural control dependent on body balance, a fundamental requirement for movement and activities of daily living; a negative consequence of decreased balance ability is an increased risk of injury. Thus, it may be more relevant to focus on functional dynamic tests [48,50] rather than isometric muscle strength testing.

A symptom of PFP is pain around or behind the patella, which is often exacerbated by loading of the patellofemoral joint in a flexed-knee position. A 4-week (12 sessions) anodal tDCS applied to M1 combined with open kinetic chain exercises was shown to significantly increase the muscular strength in women with PFP [51]. Ho et al. [52] provided some preliminary insights into the acute effects of 20 min bimodal tDCS over M1s paired with resistance exercise on individuals with PFP. No improvements in frontal plane movements or pain were observed. Once again, the combination of transcranial direct current stimulation (tDCS) and resistance training appeared to be more advantageous.

Overall, the current evidence presented in Table 1 suggests that the synergistic effects of tDCS and physical therapy/exercise, when administered repeatedly over a period of several weeks, may be beneficial for CNS functions and potentially accelerate recovery as well as enhance long-term performance. These findings, underlined before, suggest that tDCS, when used alongside traditional rehabilitation methods, may help individuals return to their pre-injury performance levels more quickly and with lower risk of reinjury. Furthermore, tDCS may help prevent disuse atrophy, a common complication following muscle injuries, by promoting greater cortical engagement during rehabilitation exercises. This combined approach could lead to a more holistic and effective recovery process, ultimately helping individuals return to their pre-injury functional levels more quickly. A central mechanism for these effects is neuroplasticity—the brain’s ability to reorganize and form new synaptic connections in response to injury, experience, or environmental changes [16]. Muscle injuries often require the brain to adapt to the altered sensory and motor feedback and reroute motor signals to compensate for damaged tissues. By stimulating the motor cortex, tDCS can promote this neuroplasticity, enhancing the brain’s capacity to reorganize motor pathways and improve muscle function. This is especially important for athletes who may suffer from long-term motor impairments due to sports injuries. Anodal tDCS has been shown to enhance neuroplasticity in the motor cortex, which is responsible for controlling voluntary movements [68]. In athletes recovering from sports injuries, neuroplastic changes induced by tDCS may allow the brain to compensate for damage to the muscles or joints more quickly. This cortical reorganization can potentially reduce the rehabilitation time and risk of reinjury by facilitating more effective and coordinated movement patterns. However, it is important to note that neuroplasticity is not always beneficial in the short-term; inappropriate reorganization can sometimes lead to maladaptive motor patterns or compensatory movements (see Section 2.1). Thus, careful integration of tDCS with physical rehabilitation is essential to maximize its benefits.

### 3.3. Pain Management Using tDCS in Musculoskeletal Injury Recovery

Pain is a common symptom of muscle injuries (see Section 2.3) and can significantly impair rehabilitation progress. tDCS has been found to be effective in modulating pain perception by altering activity in brain regions involved in pain processing. Anodal stimulation to the motor cortex, prefrontal cortex, or somatosensory cortex has been reported to reduce pain intensity, enabling athletes to engage in rehabilitation exercises more comfortably [69]. Anodal tDCS is thought to reduce pain perception by increasing the excitability of pain-inhibitory pathways in the brain. A few studies have indicated that tDCS can reduce muscle pain and improve the tolerability of rehabilitation exercises. Indeed, tDCS has been employed to manage pain in individuals diagnosed with KO or those who have undergone total knee replacement. The first evidence of improvements in functional outcomes has been documented [51,53,70]. The use of tDCS over M1 has been shown to reduce experimental pain sensitivity and increase pain inhibition in older adults suffering from knee OA [53]. It has even been demonstrated that the application of repeated sessions of anodal tDCS over M1 can achieve a similar degree of analgesia with a reduced consumption of opioids in the postoperative period following total knee arthroplasty; four sessions of tDCS over M1 could reduce morphine consumption and pain perception during the postoperative period in total knee arthroplasty [70].

Anodal tDCS applied to M1 combined with open kinetic chain exercises has been shown to reduce pain perception in women with PFP [51]. Combining tDCS with strengthening exercise in KO individuals showed reduced pain following 8-weeks of treatment and found that tDCS improved overall physical function [54]. This reduction in pain sensitivity may allow patients to engage more fully in physical therapy or strength training, which can improve the overall rehabilitation outcomes. For example, Rahimi et al. [55] found that tDCS over either M1 or PFC significantly reduced the pain levels in individuals with KO and improved functional performance tests. Wu et al. [71], in their metanalysis, noted that tDCS could reduce the short-term pain intensity and sensitivity in patients with KO, which allowed for increased participation in both physical therapy and strength training. The same conclusion was found by the meta-analysis of Yang et al. [72], but they highlighted that tDCS intervention may not have a large efficacy in physical function and mobility performance.

The PFC, when targeted by tDCS, is particularly involved in pain perception and the emotional experience of pain [45,73]. By modulating activity in this region, tDCS can potentially help alleviate both the sensory and emotional aspects of pain, making it a valuable tool for improving recovery in athletes or individuals recovering from muscle injuries. Approaches, such as closed-loop tDCS, involving real-time monitoring (e.g., via electroencephalography or functional near-infrared spectroscopy [74]) of the brain states sensitive to pain processing, could be implemented in rehabilitation therapy following musculoskeletal injuries. The evaluation of the pain processing network, encompassing the sensorimotor regions (pain intensity) and the prefrontal cortex (attention and emotional response to pain), facilitates the real-time adjustment of stimulation parameters during the tDCS session. Furthermore, when administered over M1, tDCS is hypothesized to induce changes in the cortical and subcortical brain regions involved in descending pain inhibition [75]. This global pain modulation effect from tDCS from multiple brain areas/networks appears valuable for rehabilitation, as it helps to reduce discomfort and promote a more active and consistent rehabilitation process following musculoskeletal injuries.

## 4. Discussion

tDCS shows considerable benefit as an adjunctive tool in sports injury rehabilitation. The current evidence suggests that tDCS can improve motor recovery, enhance neuroplasticity, reduce pain, and accelerate rehabilitation outcomes. The ability of tDCS to modulate cortical excitability and facilitate brain reorganization is particularly beneficial for athletes recovering from musculoskeletal injuries, where the CNS plays a critical role in functional recovery. The effectiveness of tDCS in enhancing motor function recovery and inducing long-lasting changes in the brain largely depends on careful optimization of the stimulation parameters. Despite the promising findings underlined in this narrative review, there are still several challenges and limitations associated with the use of tDCS in muscle injury rehabilitation.

While the clinical potential of tDCS in muscle injury recovery is significant, the optimal stimulation parameters (e.g., intensity, duration, frequency of sessions) remain unclear. There was considerable variability in the tDCS protocols across studies including differences in electrode placement, stimulation duration, and current intensity [76]. It is also imperative to consider the potential impact of relevant inter-individual factors on tDCS variability at the intra-study level [77]. These inconsistencies make it difficult to draw definitive conclusions about the optimal parameters for tDCS treatment in muscle injury recovery. Further research is needed to determine the most effective protocols and to establish clear guidelines for tDCS application in different types of muscle injuries. The combination of tDCS with motor training, physical therapy, and personalized protocols could significantly increase the likelihood of inducing sustained improvements in motor control and brain network reorganization.

Additionally, the individual variability in response to tDCS poses a challenge. Factors such as age, injury severity, and baseline motor function may influence the effectiveness of tDCS, necessitating personalized treatment protocols. As posited by [78], a precision approach with tES techniques would enable the tailoring of both the individual neuroanatomy through structural imaging and computer modeling as well as the temporal characteristics of stimulation with regard to endogenous brain oscillatory activity. This would potentially maximize the efficacy of brain stimulation with personalized dosing. Further research into individualized approaches and long-term monitoring will be essential for refining the tDCS technique and ensuring its clinical efficacy in muscle injury rehabilitation.

Furthermore, while tDCS may offer short-term benefits, the long-term effects on motor recovery and reinjury prevention remain unclear. Longitudinal studies with larger sample sizes are needed to assess the sustained impact of tDCS on muscle injury recovery.

## 5. Conclusions

Musculoskeletal injuries can have profound and lasting effects on the CNS. These effects range from altered motor control and neural activation, increased inhibition of the injured muscle, and changes in neuroplasticity to changes in pain perception. While the brain can reorganize and adapt to these changes, prolonged or maladaptive adaptations may result in chronic dysfunction. The CNS plays a critical role in both the immediate response to injury and the long-term recovery process, so effective rehabilitation strategies that address not only the injured muscle, but also the neural components of recovery, are crucial. Interventions like neuroplasticity-based rehabilitation, proprioception training, and pain management are important for restoring optimal neural function and muscle performance after a muscle injury.

Non-invasive brain stimulation, specifically tDCS, represents a promising non-invasive tool for enhancing brain function and accelerating recovery after muscle injuries. By facilitating motor recovery, modulating pain, and promoting neuroplasticity, tDCS can accelerate rehabilitation processes and improve functional outcomes. As the field continues to evolve, tDCS may become an integral part of rehabilitation programs for individuals recovering from musculoskeletal injuries, particularly in athletes who require fast recovery and optimal functional performance. Future studies should focus on determining the most effective treatment protocols, exploring the long-term benefits, and refining our understanding of how tDCS can be integrated into comprehensive sports injury rehabilitation regimens.

## Figures and Tables

**Table 1:** Summary of studies (original article/investigation) using tDCS intervention in people/individuals who had experienced common musculoskeletal injuries (CAI, ACL, KO, and PFP).

Type of Injury Author	Aim	Study Design Intervention tDCS Participants	Outcomes Measures Testing	Main Findings Beyond Those of Sham
CAI Bruce et al. [46]	Conduct a preliminary investigation into the feasibility and efficacy of an intervention of eccentric ankle exercise in conjunction with tDCS in improving cortical excitability, functional performance, and patient-reported outcomes	Longitudinal randomized, single-blinded and sham-controlled 4-weeks eccentric ankle strength training (60% maximal torque)-tDCS Anodal tDCS (1.5 mA, 18 min) over M1 22 adults (18–40 years, 17 females): 2 groups of 11	Functional performance, strength Cortical excitability, inhibition Dynamic balance, muscle activation	↑ M1 excitability, dynamic postural stability, muscle recruitment during a hop- to-stabilization, and ↓ perceived disablement with tDCS in conjunction with eccentric training (extra effect) Improvements most notable at the retention +2 weeks
CAI Huang et al. [47]	Investigate the effects of tDCS combined with Bosu ball training on the injury during drop landing	Single-blinded and sham-controlled 18 sessions Bosu ball training barefoot-tDCS, 6 weeks (3/week) Anodal tDCS (2 mA, 20 min) over M1 34 adults (20.5 years, tDCS + Bosu: 18)	Drop landing test (peak ankle inversion angle, velocity, and time to peak)	↓ peak ankle inversion angular velocity and the plantar flexion angle at the peak angle inversion for combined tDCS + Bosu ball (extra effect)
CAI Ma et al. [48]	Examine the effects of an intervention comprising short-foot exercise combined with high definition tDCS on proprioception and dynamic balance	Double-blinded and sham-controlled 12 sessions short-foot exercise-tDCS, 4-weeks (3/week) Anodal tDCS (2 mA, 20 min) over M1 28 adults (18–30 years, 15 females): 2 groups of 14	Ankle inversion judgement, joint position perception, Y balance test, sensory organization test equilibrium score	↑ performance (Y-balance test, the sensory organization test, and the joint position test with the ankle at 15° inversion); extra effects of exercise combined with tDCS
ACL Rush et al. [49]	To determine if a single treatment of tDCS would improve quadriceps muscle activity and reduce self-reported levels of pain during exercise	Randomized Crossover tDCS during Walking (2.0 mph 1% incline) tDCS Halo unit (around 1.1 mA, 20 min) over M1 10 adults (23 years, 5 females)	Quadriceps: maximal voluntary isometric contraction (MVIC), central activation ratio (CAR), muscle EMG, vasti Knee Injury Osteoarthritis Outcome Score (KOOS)	No differences in quadriceps MVIC and CAR or KOOS when comparing active tDCS and sham
ACL Murphy et al. [11]	To determine if anodal tDCS can alter quadriceps intracortical inhibition and facilitation in an ACL population after 6 weeks of application during exercise (from week 2 post ACL)	Randomized, triple-blind controlled trial 18 sessions tDCS, 6 weeks (3/week), from week 2 add-on exercise rehabilitation (20–60 min) Anodal tDCS (2 mA, 20 min) over M1 21 adults (24 years, 8 females, anodal group: 11)	Quadriceps/hamstring: short-interval intracortical inhibition (SICI), long-interval intracortical inhibition (LICI), short-interval intracortical facilitation (SICF) Quadriceps MVIC	Benefit for the tDCS group on the muscle function: Quadriceps ↓ SICI and SICF No changes MVIC, LICI Hamstring ↓ SICF No changes SICI, LICI and SICF
ACL Tohidirad et al. [50]	To examine the effects of anodal tDCS over (M1) concurrent with physiotherapy training (PT) on postural control and muscular performance	Randomized, single-blinded and sham-controlled 10 sessions tDCS with PT. Anodal tDCS (2 mA, 20 min) over M1 34 adults (anodal with PT group: 16)	Center of pressure displacement Power of flexors and extensors	Benefit for the tDCS group on the postural control: one month after, ↓ center of pressure, ↑ power flexors and extensors
PFP Rodrigues et al. [51]	To investigate the effects of tDCS applied to M1 combined with knee exercises on muscular strength and pain perception	Counterbalanced crossover 12 sessions Resistance Training (RT) protocol: 60% 10 RM, knee open kinetic chain Anodal tDCS (2 mA, 20 min) over M1 before each session 28 women (23 years)	10 repetition maximum (RM) test Pain perception	↑ 10 RM load (strength gain) in tDCS + RT no differences for pain perception between groups but lower after intervention in tDCS + RT
PFP Ho et al. [52]	To explore whether single session of bimodal tDCS over M1 paired with exercise could alleviate PFP and enhance frontal plane kinematics in the lower extremity and trunk	Double-blinded, sham-controlled crossover Resistance exercises (ankle weight at 30% RM) during tDCS Anodal tDCS (2 mA, 19 min) over M1s 10 adults (28.2 years, 4 females)	Frontal plan movements (video) during 5 weight-bearing tests: trunk lean angle, knee frontal plane projection angle, and dynamic valgus index Pain visual analog scale	No improvement in frontal place movements or pains
KO Ahn et al. [53]	To examine the effect of tDCS on experimental pain sensitivity in older adults with KO and how these changes in experimental pain sensitivity are related to KO-related clinical pain and function changes	Data 5 consecutive days Anodal tDCS (2 mA, 20 min) over M1 40 adults (50–70 years, 21 females); two groups of 20	Multimodal quantitative sensory testing battery (heat pain, pressure pain threshold, punctate mechanical pain, and conditioned pain modulation)	Active tDCS group: ↑ heat pain thresholds and tolerances pressure pain threshold and conditioned pain modulation ↓ clinical pain
KO Chang et al. [54]	To assess the safety and feasibility of adding tDCS to quadriceps strengthening exercise	Randomized, participant-blinded controlled trial 8 weeks (16 sessions, 2/week) Anodal tDCS (1 mA, 20 min) prior 30 min of supervised strengthening exercise (ankle cuff weights/resistance bands) 30 adults (60–64 years, 20 females, 2 groups of 15)	Pain (visual analog scale), function (questionnaire) and perceived effect (Likert scale) Pain mechanism (thresholds, reflex)	Adding tDCS to strengthening exercise: ↑ pain function and pain mechanisms
KO Rahimi et al. [55]	To investigate the effect of adding tDCS to conventional PT on pain and performance	Randomized, double-blind clinal trial 10 sessions (5/week) of PT (6 exercises) Anodal tDCS (1 mA, 20 min) over left M1, left sensorimotor cortex or left PFC 80 adults (58.8 years, 72 females)	Visual analogue scale (VAS) for pain intensity KOOS questionnaire Range of motion knee flexion 10-min walking test	Adding tDCS to PT: ↑ pain and physical performance

↑ = increase, ↓ = decrease.

## Abbreviations

The following abbreviations were used in this manuscript:

ACL	Anterior cruciate ligament
CAI	Chronic ankle instability
CNS	Central nervous system
CAR	Central activation ratio
KO	Knee osteoarthritis
EMG	Electromyography
M1	Primary motor cortex
KOOS	Knee Injury Osteoarthritis Outcome Score
LICI	Long-interval intracortical inhibition
MVIC	Maximal voluntary isometric contraction
PFC	Prefrontal cortex
PFP	Patellofemoral pain
PT	Physiotherapy training
RM	Repetition maximum
RT	Resistance training
SICI	Short-interval intracortical inhibition
SICF	Short-interval intracortical facilitation
tDCS	Transcranial direct current stimulation
tES	Transcranial electrical stimulation
